# Validation of albumin platelet product as a non-invasive fibrosis staging tool in patients with chronic HCV-related liver disease

**DOI:** 10.1038/s41598-025-07456-x

**Published:** 2025-06-23

**Authors:** Maha Elsabaawy, Mohamed Eissa, Ahmed Shaban, Madiha Naguib

**Affiliations:** Department of Hepatology and Gastroenterology, National Liver Institute, Menoufia University, Shebeen Elkoom, Menoufia, Egypt

**Keywords:** Albumin platelet product (APP), Fibrosis, Hepatitis C virus (HCV), Non-invasive fibrosis markers, Biomarkers, Gastroenterology

## Abstract

Background and aim: The Albumin Platelet Product (APP) has emerged as a promising non-invasive biomarker for fibrosis staging in chronic liver disease (CLD). This cross-sectional study aims to evaluate the effectiveness of APP compared to established non-invasive markers of fibrosis in an Egyptian cohort with HCV-related CLD. Methods: 580 participants were assessed across different fibrosis stages (F0-F4) to analyze the relationship between APP and liver fibrosis. APP was compared with FIB-4 and APRI scores for diagnostic performance. Results: The study included 580 patients with HCV-related CLD (mean age: 37.6 ± 9.66 years; 74.3% males). APP proved superiority in identifying liver cirrhosis (F4) at Cut-off values ≤ 0.59 with 81% sensitivity, 63.6% specificity (*p* < 0.001). APP showed a significant correlation with fibrosis stages, with an AUC of 0.920 (95% CI: 0.888–0.953) for distinguishing F4 from F0-F3 surpassing both FIB4 and APRI scores. However, FIB-4 proved superiority in distinguishing advanced fibrosis (F ≥ 3) with AUC of 0.899 (95% CI: 0.871–0.927) compared to APP with AUC of 0.87 (95% CI: 0.84–0.90), respectively. Multivariate analysis confirmed APP as an independent predictor of fibrosis (OR: 0.997, 95% CI: 0.995–0.998; *p* < 0.001). Conclusion: APP showed the highest performance in predicting cirrhosis, suggesting its potential as a simple, non-invasive marker for identifying patients with advanced liver disease. Its integration into clinical practice may enhance early detection and risk stratification in chronic HCV-related fibrosis. However, further multicenter, longitudinal studies are required to validate its efficacy across diverse populations and other liver disease etiologies.

## Introduction

 Chronic hepatitis C virus (HCV) infection remains a major global cause of liver fibrosis and cirrhosis. In addition to direct hepatic injury, HCV exerts systemic metabolic effects, most notably through the induction of insulin resistance via disruption of insulin signaling pathways^1^. This metabolic derangement, often occurring early in the course of infection, contributes to hepatic steatosis and may accelerate fibrogenesis independently of viral load or inflammation^2^. These multifactorial drivers of fibrosis underscore the need for accurate, non-invasive tools to stage liver fibrosis in HCV-infected patients^3^.

Among the commonly used non-invasive indices, the Fibrosis-4 index (FIB- and the Aspartate Aminotransferase to Platelet Ratio Index (APRI) are simple and widely adopted serological markers in chronic HCV^4^. However, both have important limitations. FIB-4 is influenced by patient age and hepatitis activity grade, which may skew results, especially in younger populations or those with fluctuating transaminase levels^5^. APRI, similarly, may overestimate fibrosis due to AST elevation from necroinflammatory activity rather than fibrosis alone^4,5^.

In response to these limitations, the Albumin Platelet Product (APP) was introduced by Fujita et al. in 2021 as a novel, simple, and reproducible index for fibrosis assessment. APP is calculated using only serum albumin and platelet count, two routine and inexpensive laboratory parameters, and—crucially—is unaffected by age or inflammatory activity grade^6^. This age-independence and simplicity make APP a particularly attractive tool for use in resource-limited settings and in patient groups where other markers may be unreliable.

This study aims to evaluate whether the Albumin Platelet Product (APP) can serve as a reliable non-invasive fibrosis marker in patients with chronic HCV-related liver disease. Specifically, the research investigates whether APP functions as a complementary tool to existing indices—such as FIB-4 and APRI—or potentially as a standalone alternative in settings where other tools may be less applicable. The primary outcome is the diagnostic accuracy of APP in identifying advanced liver fibrosis (F ≥ 3), using liver biopsy as the reference standard.

## Patients and methods

This monocentric, cross-sectional observational study was conducted at the National Liver Institute, Menoufia University. All methods were carried out in accordance with relevant guidelines and regulations.

### Patient selection

**Fig. 1:**
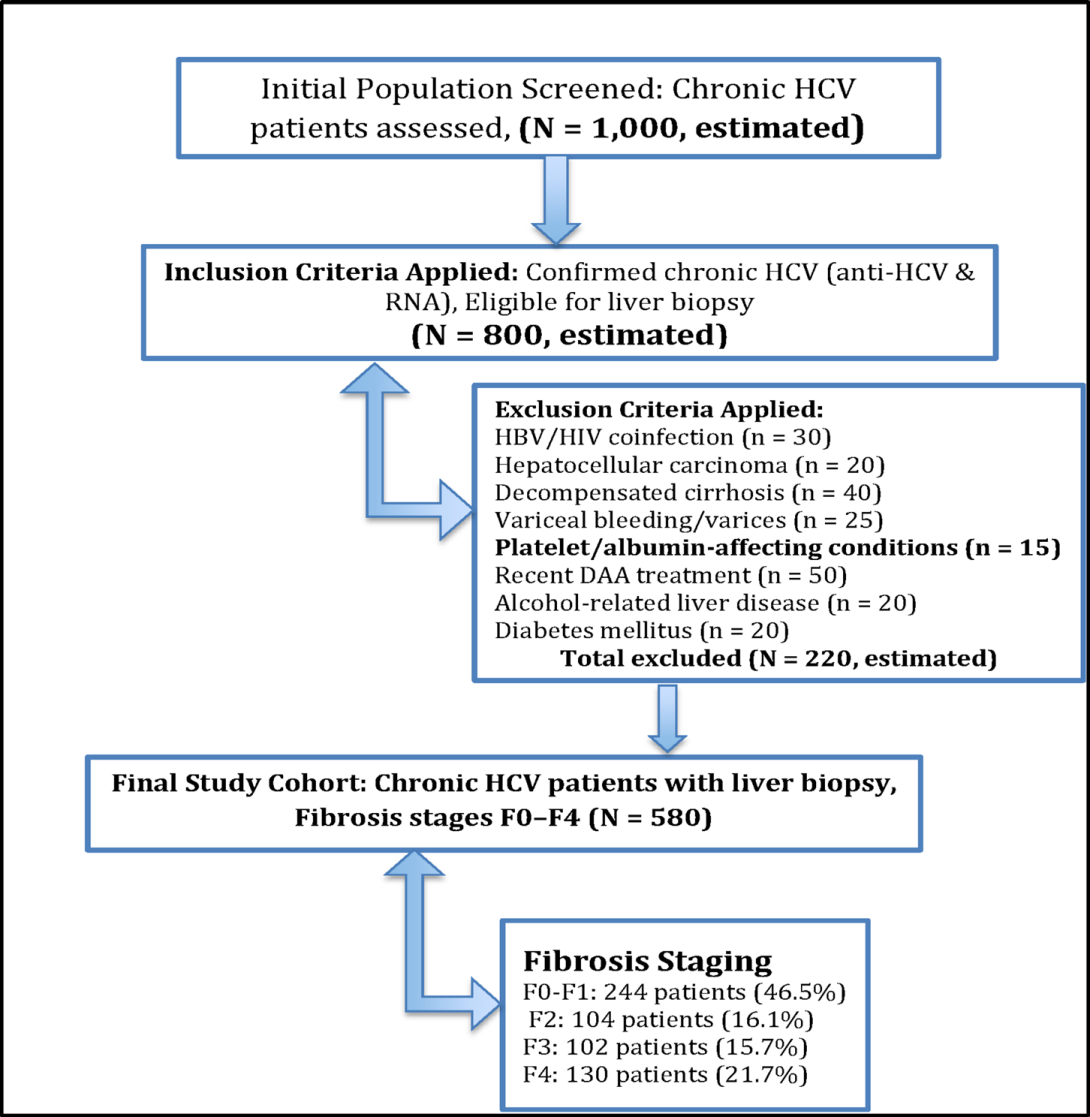
Patient Selection Flow Diagram.

The study included 580 patients diagnosed with chronic HCV infection (Fig. 1).

### Exclusion criteria

- Coinfection with HBV or HIV.
- Hepatocellular carcinoma (HCC).
- Decompensated liver cirrhosis.
- Active variceal bleeding or esophageal varices.
- Chronic conditions affecting platelet count or albumin levels (e.g., hematologic disorders).
- Recent treatment with direct-acting antivirals (DAAs).
- Alcohol-related liver disease.
- Diabetes mellitus (due to potential confounding effects on fibrosis progression).

## Study protocol

The following clinical data were recruited: age, sex, liver tests, and complete blood count. For diagnosis of HCV infection, anti-HCV antibodies were enrolled and were further evaluated for quantitative HCV RNA. All eligible patients underwent percutaneous liver biopsy under real-time ultrasound guidance after obtaining informed consent. A single pass was performed using a 14-gauge Tru-Cut needle.

### Histopathological evaluation

Biopsy specimens containing at least five portal tracts were deemed adequate for histological evaluation. All samples were interpreted by a single experienced hepatopathologist who was blinded to the patients’ clinical and laboratory data to ensure unbiased fibrosis staging. A modified METAVIR score with five fibrosis stages, ranging from F0 to F4, was used to determine the amount of fibrosis as follows: F1, portal or central fibrosis without septa; F2, few septa; F3, numerous septa; and F4, cirrhosis^7^.

### Scores computation

Calculate Albumin Platelet Product (APP) using albumin and platelet count for each participant. Obtain Fibrosis-4 (FIB-4) and AST To Platelet Ratio Index (APRI) scores as comparative fibrosis markers. The scores were calculated using the following equations^6^^[,8^^[–13^:

$${\mathbf{APP}}\,=\,{\text{Albumin }}\left( {{\text{g}}/{\text{l}}} \right) \times {\text{platelet }}( \times {\text{1}}{0^{\text{9}}}/{\text{l}})/{\text{ 1}}000.$$

$${\mathbf{FIB}} - {\mathbf{4}}{\text{ }}{\mathbf{index}}\,=\,{\text{Age }}\left( {{\text{years}}} \right) \times {\text{AST }}\left( {{\text{U}}/{\text{L}}} \right)/{\text{ }}[{\text{PLT }}\left( {{\text{1}}{0^{\text{9}}}/{\text{L}}} \right) \times \surd {\text{ALT }}\left( {{\text{U}}/{\text{L}}} \right)].$$

$${\mathbf{APRI}}={\text{ }}\left[ {\left( {{\text{AST }}\left( {{\text{IU}}/{\text{L}}} \right)/{\text{Upper normal limit of AST }}\left( {{\text{IU}}/{\text{L}}} \right)} \right)/{\text{ Platelet count }}\left( {{\text{1}}{0^{\text{9}}}/{\text{L}}} \right)} \right].$$

### Outcome measures

#### Primary outcome

Correlation between APP values and liver fibrosis stages.

#### Secondary outcomes

Comparison of APP with FIB-4 and APRI in assessing fibrosis stage and simplicity of calculation.

### Statistical analysis

Data was analyzed using IBM SPSS Statistics for Windows, Version 20.0 (IBM Corp., Armonk, NY, USA). ROC curves assessed the predictive accuracy of APP, FIB-4, and APRI for fibrosis staging. Logistic regression models identified independent predictors of advanced fibrosis (F3-F4). The Hosmer-Lemeshow test confirmed model fit (*p* = 0.561). Categorical data were represented as numbers and percentages. The chi-square test was applied to compare between two groups. Alternatively, the Monte Carlo correction test was applied when more than 20% of the cells were expected to count less than 5. For continuous data, they were tested for normality by the Shapiro-Wilk test. Quantitative data were expressed as range (minimum and maximum), mean, standard deviation, and median for normally distributed quantitative variables. A one-way ANOVA test was used for comparing the four studied groups followed by a Post Hoc test (Tukey) for pairwise comparison. On the other hand, for not normally distributed quantitative variables The Kruskal Wallis test was used to compare different groups, followed by the Post Hoc test (Dunn’s for multiple comparisons test) for pairwise comparisons. The significance of the results obtained was judged at the 5% level. To account for multiple group comparisons, one-way ANOVA (with Tukey post hoc) or Kruskal–Wallis tests (with Dunn’s post hoc) were used where appropriate. Although Bonferroni correction was not applied, multiple comparisons were managed through these post hoc adjustments to mitigate type I error. For confounding control, multivariate logistic regression analysis was performed on variables found significant in univariate analysis, allowing the identification of independent predictors of advanced fibrosis.

## Results

### Demographic data of the studied patients

Five hundred-eighty patients with chronic liver disease due to HCV infection had undergone liver biopsy 74.3% were male and their mean age was 37.6 ± 9.66 years. Patients with different stages of liver fibrosis were found as follows: 244 (46.5%) patients had stage F0–F1, 104 (16.1%) patients had stage F2, 102 (15.7%) patients had stage F3, and 130 (21.7%) patients had stage F4. The median values of APP, FIB-4, and APRI scores were 0.8, 1.19, and 0.56 respectively (Tables 1a and 1b).

### Characters of patients with different liver fibrosis stages

**Fig. 2:**
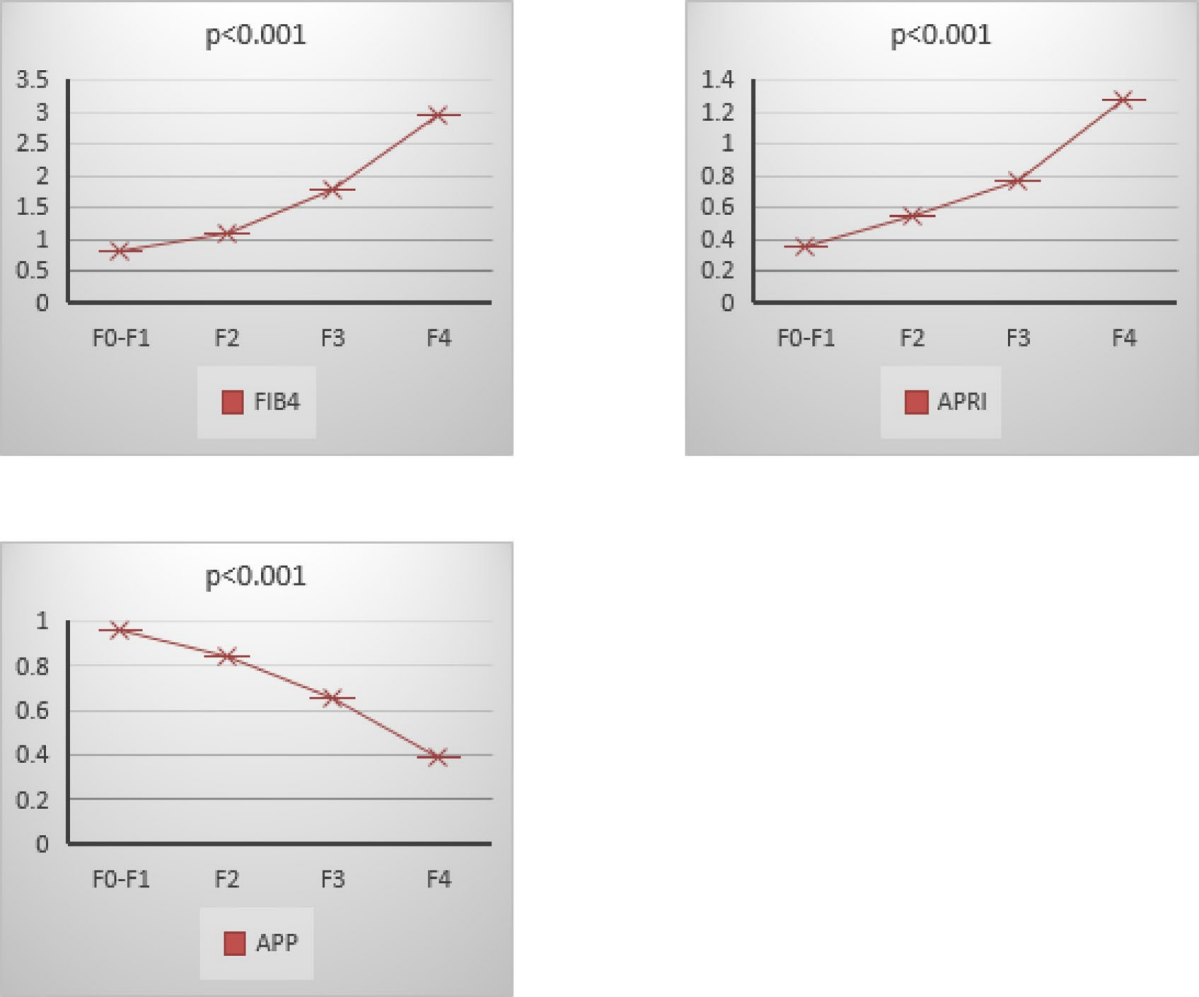
Values of FIB-4, APRI and APP in Different Fibrosis Stages.

Patients with F4 liver fibrosis tended to be older with a mean age of 46.5 ± 6.96 years, with significantly higher values of BMI, bilirubin, AST, ALT, INR, FIB-4, and APRI scores (*p* < 0.001), while total protein, albumin, Hb, platelets and APP values were significantly lower (*p* < 0.001) (Tables 2a and 2b; Fig. 2).

### Diagnostic ability of APP for liver fibrosis stages

Area under the receiver operating characteristic curve (AUROC) values were 0.640 (0.567–0.713), 0.681 (0.594–0.768), and 0.817 (0.754–0.881) (*p* < 0.001) for F2, F3, and F4 respectively (Table 3). The ROC analysis demonstrated that APP score was able to predict liver cirrhosis ≥ F4 satisfactorily at Cut-off values ≤ 0.59 with 81% sensitivity, 63.6% specificity, and 75.7 positive likelihood ratio (Table 3).

### Comparison between APP, FIB-4 and APRI in the studied patients

**Fig. 3:**
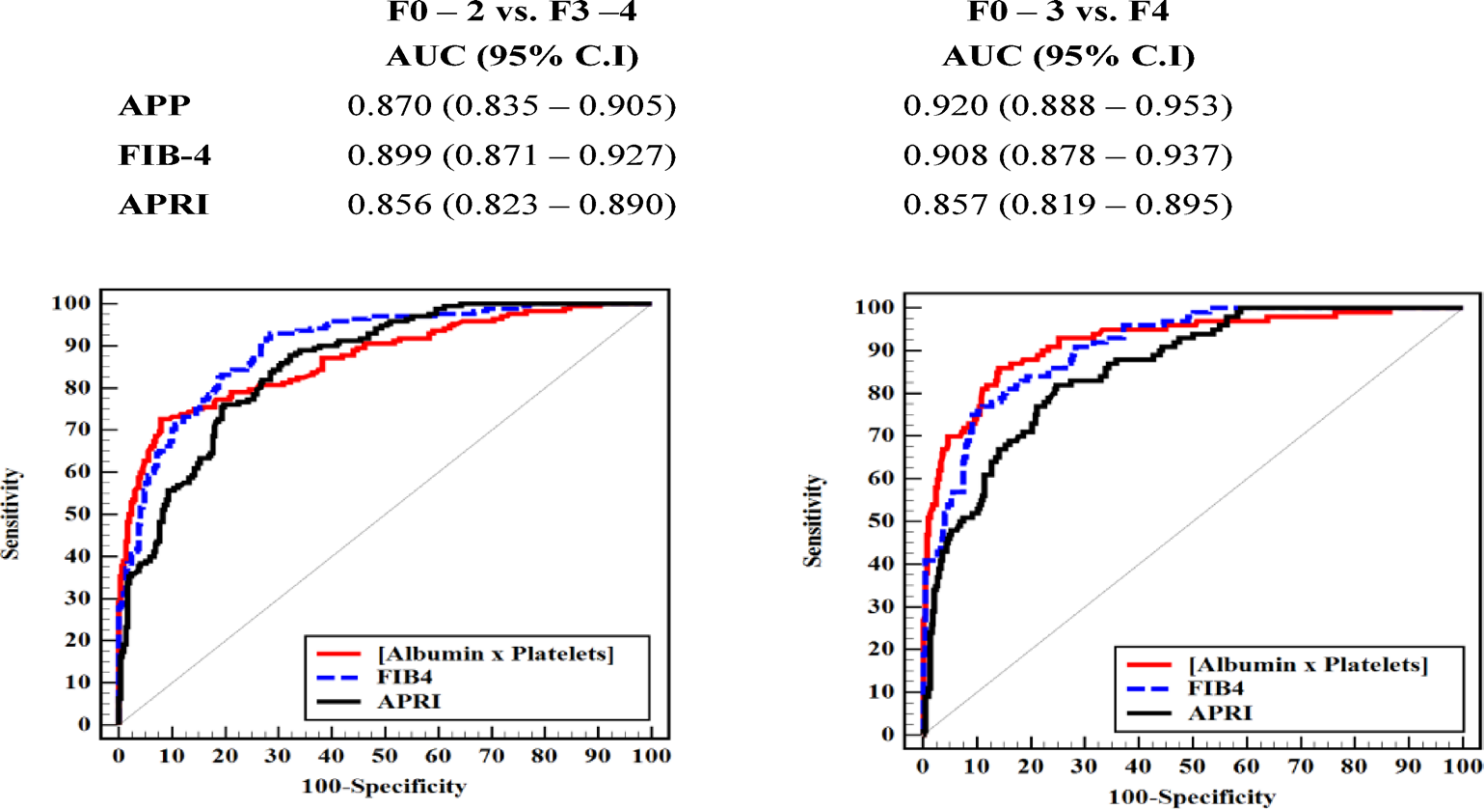
**a**: ROC curve to discriminate advanced (F3–F4) from non-advanced fibrosis (F0–F2) (*n* = 348 vs. 232). **b**: ROC curve to discriminate cirrhotic (F4) from non-cirrhotic (F0–F3) (*n* = 130 vs. 450).

Regarding to distinguish non-advanced fibrosis (F ≤ 2) from advanced fibrosis (F ≥ 3), FIB-4 showed the highest AUROC then APP and APRI 0.899 (95% CI: 0.871–0.927), 0.87 (95% CI: 0.84–0.90) and 0.856 (95% CI: 0.823–0.890) respectively (Fig. 3a).

As to differentiate between cirrhosis (F ≥ 4) and non-cirrhosis stages (F < 4), AUROC was higher in APP and FIB4 0.920 (95% CI: 0.888–0.953) and 0.908 (95% CI: 0.878–0.937) respectively, while AUROC for APRI was 0.857 (95% CI: 0.819–0.895) remained smaller than the rest (Fig. 3b).

### A logistic regression analysis for prediction of advanced liver fibrosis

By univariate logistic regression, the following variables were statistically independent predictors for advanced fibrosis ≥ F3: age, total and direct bilirubin, liver enzymes, PT, INR, CBC, FIB-4, APRI and APP. On multivariate analysis only Age, PT and APP were positively correlated with the presence of advanced liver fibrosis (*p* = 0.029, 0.001 and < 0.001) respectively (Table 4).

**Table 1a:** The whole cohort Demographics.

Parameters	Median (IQR)/Number (%)
Age (years)	37.6 ± 9.66
Sex	
Male	342 (74.3%)
Female	118 (25.7%)
BMI (kg/m^2^)	26.7 ± 2.72

BMI, body mass index; IQR, interquartile range.

**Table 1b:** The whole cohort biochemical and fibrosis parameters (*n* = 580).

Parameters	Median (IQR)/Number (%)
Albumin (g/l)	4.23 ± 0.51
AST (U/L)	48.7 ± 31.4
ALT (U/L)	53 ± 35.2
ALP (U/L)	109.00 ± 34.13
P.T %	85.8 ± 11.0
INR	1.12 ± 0.15
Hb (G/DL)	13.32 ± 1.67
WBCs ×10³/µL	6.33 ± 6.58
Platelets (10^9^×L)	185.6 ± 63.97
Fibrosis stages (LP)	
F0 – F1	(*n* = 244)
F2	(*n* = 104)
F3	(*n* = 102)
F4	(*n* = 130)
Non-invasive fibrosis markers	
APP	0.81 (0.02–1.74)
FIB-4	1.19 (0.26–71.4)
APRI	0.56 (0.10–36.54)

BMI, body mass index; T.Bil; total bilirubin; D. Bil, direct bilirubin; AST, aspartate aminotransferase; ALT, alanine aminotransferase; ALP, alkaline phosphatase; PT, prothrombin time; INR, international normalized ratio; Hb, hemoglobin; WBCs, white blood cells, IQR, interquartile range; LP: Liver biopsy; APP, albumin platelet product; FIB-4, fibrosis-4; APRI, AST to platelets ratio index.

**Table 2a:** Comparison between the different studied groups of liver fibrosis according to demographic parameters.

	Liver biopsy				Test of Sig.	*p*
Mean ± SD.	F0 – F1 (*n* = 244)	F2 (*n* = 104)	F3 (*n* = 102)	F4 (*n* = 130)		
Age (years)	33.5 ± 8.85	35.3 ± 8.14	39.8 ± 7.67	46.5 ± 6.96	F = 61.603^*^	< 0.001^*^
Male Sex	188 (77.1%)	75 (71.6%)	77 (75.0%)	91 (70.0%)	χ2 = 2.147	0.542
BMI (kg/m^2^)	26.5 ± 2.88	26 ± 2.86	26.9 ± 2.82	27.7 ± 1.81	H = 19.445^*^	< 0.001^*^

BMI, body mass indexSD: Standard deviation, χ^2^: Chi-square test, F: F for One way ANOVA test, Pairwise comparison bet. each 2 groups were done using Post Hoc Test (Tukey), H: H for Kruskal Wallis test, and Pairwise comparison bet. each 2 groups were done using a Post Hoc Test (Dunn’s for multiple comparisons test), p: p-value for comparing between the different studied groups *: Statistically significant at *p* ≤ 0.05.

**Table 2b:** Comparison between the different studied groups of liver fibrosis according to biochemical and fibrosis parameters.

	Liver biopsy				Test of Sig.	*p*
Mean ± SD.	F0 – F1 (*n* = 244)	F2 (*n* = 104)	F3 (*n* = 102)	F4 (*n* = 130)		
T. Bil (µmol/l)	0.71 ± 0.25	0.72 ± 0.26	0.87 ± 0.31	1.54 ± 0.81	103.749^*^	> 0.001^*^
D. Bil (µmol/l)	0.26 ± 0.17	0.31 ± 0.21	0.40 ± 0.26	0.65 ± 0.41	85.281^*^	< 0.001^*^
T. Protein (g/l)	7.26 ± 0.31	7.08 ± 0.21	7.02 ± 0.28	6.61 ± 0.76	51.283^*^	< 0.001^*^
Albumin (g/l)	4.50 ± 0.33	4.38 ± 0.33	4.12 ± 0.33	3.63 ± 0.52	190.031^*^	> 0.001^*^
AST (U/L)	37.5 ± 24.6	46.3 ± 25.4	62.2 ± 38.3	64.9 ± 32.7	102.500^*^	< 0.001^*^
ALT (U/L)	42.9 ± 30	54.9 ± 34.6	63 ± 37.9	65.8 ± 38.1	58.070^*^	< 0.001^*^
ALP U/L)	111.7 ± 37.3	115.5 ± 35.79	103.82 ± 23.97	101.94 ± 30.3	10.462^*^	0.015^*^
P.T %	90.8 ± 7.87	88.8 ± 7.11	86.3 ± 7.73	72.6 ± 10.6	111.589^*^	< 0.001^*^
INR	1.05 ± 0.12	1.09 ± 0.11	1.14 ± 0.11	1.28 ± 0.15	81.830*	< 0.001*
Hb (g/Dl)	13.69 ± 1.50	13.51 ± 1.37	13.67 ± 1.56	12.14 ± 1.78	24.962*	< 0.001*
WBCs^3^×10/µL	6.70 ± 5.28	6.24 ± 1.96	7.32 ± 13.55	4.89 ± 1.71	41.487*	< 0.001*
Platelets (10^9^×L)	218.1 ± 53.69	196.6 ± 50.33	169.6 ± 54.11	119.5 ± 42.79	182.125^*^	> 0.001^*^

T-bill; total bilirubin; D. Bil, direct bilirubin; AST, aspartate aminotransferase; ALT, alanine aminotransferase; ALP, alkaline phosphatase; PT, prothrombin time; INR, international normalized ratio; Hb, hemoglobin; WBCs, white blood cells, SD: Standard deviation, χ^2^: Chi-square test, F: F for One way ANOVA test, Pairwise comparison bet. each 2 groups were done using Post Hoc Test (Tukey), H: H for Kruskal Wallis test, and Pairwise comparison bet. each 2 groups were done using a Post Hoc Test (Dunn’s for multiple comparisons test), p: p-value for comparing between the different studied groups *: Statistically significant at *p* ≤ 0.05.

**Table 3:** Diagnostic performance for APP to predict liver biopsy stage.

	AUC	*p*	95% C. I	Cut off	Sensitivity	Specificity	PPV	NPV
F2	0.640	< 0.001^*^	0.567–0.713	≤ 0.94	70.27	52.80	34.0	83.7
F3	0.681	< 0.001^*^	0.594–0.768	≤ 0.83	65.28	50.00	56.0	59.7
F4	0.817	< 0.001^*^	0.754–0.881	≤ 0.59	81.0	63.89	75.7	70.8

AUC: Area Under a Curve, p-value: Probability value, CI: Confidence Intervals, NPV: Negative predictive value, PPV: Positive predictive value, *: Statistically significant at *p* ≤ 0.05.

**Table 4:** Univariate and multivariate logistic regression analysis for the different parameters affecting advanced liver fibrosis (F3 – F4).

	Univariate		^#^Multivariate	
	*p*	OR (LL – UL 95%C. I)	*p*	OR (LL – UL 95%C. I)
Age (years)	< 0.001^*^	1.135 (1.105–1.165)	0.029^*^	1.063 (1.006–1.123)
Female	0.392	1.206 (0.785–1.850)		
BMI (kg/m^2^)	< 0.001^*^	1.153 (1.072–1.241)	0.331	0.937 (0.823–1.068)
T-bil (µmol/l)	< 0.001^*^	16.236 (8.412–31.338)		
D-Bil (µmol/l)	< 0.001^*^	41.812(17.170 − 101.819)		
Total Protein (g/l)	< 0.001^*^	0.059 (0.029–0.123)		
AST (U/L)	< 0.001^*^	1.032 (1.023–1.041)		
ALT (U/L)	< 0.001^*^	1.016 (1.010–1.023)		
ALP (U/L)	0.003^*^	0.991 (0.985–0.997)		
Prothrombin %	< 0.001^*^	0.879 (0.856–0.902)	0.001^*^	0.943 (0.911–0.976)
INR^$^	< 0.001^*^	2.401 (2.004–2.875)		
Hb (g/Dl)	< 0.001^*^	0.721 (0.639–0.815)		
WBCs ×10³/µL	0.362	0.972 (0.916–1.033)		
Albumin (g/l)	< 0.001^*^	0.021 (0.010–0.044)		
Platelets (10^9^×L)	< 0.001^*^	0.973 (0.967–0.978)		
APP	< 0.001^*^	0.993 (0.992–0.995)	< 0.001^*^	0.997 (0.995–0.998)
FIB-4	< 0.001^*^	7.372 (4.980–10.914)	0.159	1.975 (0.766–5.089)
APRI	< 0.001^*^	15.057 (8.389–27.026)	0.545	1.434 (0.446–4.609)

BMI, body mass index; T-bil; total bilirubin; D-Bil, direct bilirubin; AST, aspartate aminotransferase; ALT, alanine aminotransferase; ALP, alkaline phosphatase; PT, prothrombin time; INR, international normalized ratio; Hb, hemoglobin; WBCs, white blood cells, Hosmer and Lemeshow Test (χ^2^ = 6.772; *p* = 0.561) OR: Odd`s ratio C.I: Confidence interval LL: Lower limit UL: Upper Limit, #: All variables with *p* < 0.05 were included in the multivariate, *: Statistically significant at *p* ≤ 0.05 $: for each 0.1.

## Discussion

Noninvasive assessments of liver fibrosis are currently an essential part of the standard of care for patients with suspected or confirmed hepatic affection. So, the researchers have expanded their efforts in disease management and have paid much attention to the evaluation of novel talented markers^14–16^.

This study substantiated APP as a promising non-invasive fibrosis staging tool in HCV-related chronic liver disease, providing an alternative to invasive liver biopsy and other non-invasive indices like the FIB-4 index and APRI score. The analysis revealed significant correlations between APP levels and various stages of liver fibrosis, underscoring its utility and potential clinical application, particularly in regions with limited access to advanced diagnostic tools.

APP exhibited a marked decrease in value as fibrosis severity increased, with significant statistical differences observed between early (F0-F1) and advanced fibrosis stages (F3-F4) indicates its potential reliability for stratifying fibrosis severity in CLD patients. The progressive decline in serum albumin and platelet count mirrors hepatic synthetic dysfunction and portal hypertension, respectively, both hallmark features of cirrhosis^17^. Since albumin is the main form of protein circulating in the blood yet only produced by the liver and regarding low platelet count, the inflammatory process caused by HCV leads to the platelet’s consumption by the spleen. Moreover, thrombocytopenia may directly result from liver injury due to the impairment of thrombopoietin production^18^.

APP cut-off value of ≤ 0.59 for identifying cirrhosis (F4) demonstrates a high sensitivity (81%) but only modest specificity (63.6%), which carries important clinical implications. The relatively high sensitivity ensures that most patients with cirrhosis are correctly identified, minimizing the risk of underdiagnosis. However, the modest specificity indicates a considerable rate of false positives, meaning a notable proportion of patients without cirrhosis may be misclassified as cirrhotic. This could lead to unnecessary anxiety, unwarranted clinical surveillance, or even inappropriate therapeutic interventions. Conversely, the 19% false-negative rate suggests that a subset of cirrhotic patients might be missed, potentially delaying appropriate management, including screening for hepatocellular carcinoma and esophageal varices. Therefore, while APP is a valuable and accessible tool—particularly in resource-limited settings due to its reliance on simple, routinely available parameters like albumin and platelet count—it is best utilized as part of a broader non-invasive diagnostic algorithm. When used in conjunction with other fibrosis markers such as FIB-4 or APRI, APP may enhance overall diagnostic accuracy, helping to balance sensitivity and specificity. This complementary use can improve the clinical decision-making process by identifying high-risk patients who may benefit from further confirmatory testing such as transient elastography or liver biopsy.

Notably, our study showed that APP predicted cirrhosis with the highest AUC (0.920) surpassing both FIB4 and APRI scores, while FIB-4 better predicted advanced fibrosis with the highest AUC (0.899). In alignment with our findings, Fujita et al. originally introduced APP and demonstrated its significant diagnostic accuracy for fibrosis staging in chronic liver diseases, particularly in identifying cirrhosis with an AUC of 0.88. Their study also emphasized the age-independence and simplicity of APP, supporting its applicability across a wide patient spectrum [6].

This superior performance of APP in defining cirrhosis may be attributed to the fact that it combines markers of liver synthetic function (albumin) and portal hypertension (platelet count), two key features typically associated with cirrhosis^19^. On the other hand, the components of FIB-4—age, AST, ALT, and platelet count—may offer a broader reflection of hepatic inflammation and progressive fibrosis, making it particularly effective for detecting advanced but not necessarily cirrhotic disease^20^. These results highlight the potential complementary roles of FIB-4 and APP in staging liver fibrosis, with APP showing strength in detecting the most severe stage of liver damage.

In multivariate logistic regression analysis, APP emerged as an independent predictor of advanced fibrosis (F3–F4) with an odds ratio (OR) of 0.997 (95% CI: 0.995–0.998; *P* < 0.001), alongside age and prothrombin time, suggesting its robust association with fibrosis severity. The OR of 0.997 indicates a modest incremental effect, where each unit increase in APP slightly reduces the odds of advanced fibrosis, reflecting its continuous nature in the model. However, the clinical strength of APP lies in its application as a categorical diagnostic tool, particularly at a cut-off value of ≤ 0.59, which yielded high sensitivity (81%) and moderate specificity (63.6%) for detecting cirrhosis (F4), outperforming FIB-4 and APRI in this context.

APP demonstrates a superior diagnostic utility compared to the FIB-4 and APRI in specific clinical contexts, particularly for detecting cirrhosis (F4), where it exhibits exceptional accuracy with an area under the receiver operating characteristic curve (AUROC) of 0.920, surpassing FIB-4 (0.908) and APRI (0.857) making it a valuable tool for identifying at-risk patients in resource-limited settings where its reliance on simple, cost-effective parameters enhances accessibility. While its moderate specificity (63.6%) precludes APP as a standalone replacement for liver biopsy, which remains essential for precise fibrosis staging, it can effectively prioritize patients for confirmatory diagnostics like transient elastography^21^. Its age-independent formulation renders APP particularly advantageous in younger populations, where FIB-4’s inclusion of age may lead to underestimation of fibrosis severity^22^. In resource-constrained settings, APP’s reliance on readily available and cost-effective parameters—albumin and platelet count—enhances its practicality over FIB-4 and APRI, which necessitate transaminases and more complex computations^23^. Moreover, APP’s independence from inflammatory markers ensures robustness in patients with fluctuating liver enzyme profiles, where transaminase-based scores like FIB-4 and APRI may be confounded by necroinflammatory activity, making APP a preferred tool for identifying patients requiring urgent surveillance for complications such as hepatocellular carcinoma or esophageal varices^24^. Thus, APP is best integrated into a diagnostic algorithm alongside FIB-4 and APRI to optimize early cirrhosis detection and streamline referral pathways.

This study serves as the first external validation of the Albumin Platelet Product (APP) following its introduction by Fujita et al., who reported its utility in staging liver fibrosis across various chronic liver disease etiologies. Our findings confirm and extend these results in an independent, histologically validated HCV cohort. In comparative analyses, APP demonstrated superior diagnostic accuracy for cirrhosis relative to FIB-4 and APRI, aligning with Fujita’s original observations and reinforcing its potential value in clinical practice. While few studies have assessed APP beyond the original publication, our work contributes important external evidence supporting its role, particularly in resource-limited settings where accessible, non-invasive tools are essential.

These findings should be interpreted in the context of certain limitations. The cross-sectional design limits the ability to assess temporal relationships or causality. While this study serves as an external validation of the APP score in an independent HCV-infected population, further validation across broader and more diverse cohorts is warranted. In particular, the focus on a single-center HCV-only cohort may limit generalizability to other liver disease etiologies such as NAFLD/MASLD, alcoholic liver disease, or HBV. Additionally, the exclusion of patients with diabetes mellitus—despite its high prevalence in chronic liver disease—may limit applicability to routine clinical practice. Future prospective, multicenter studies that include metabolically diverse populations are needed to confirm the diagnostic utility of APP in real-world settings.

## Conclusion

APP, owing to its simplicity and reliance on routinely available laboratory parameters, may serve as an accessible adjunct in non-invasive fibrosis assessment, especially in settings lacking advanced technologies. When used in combination with established markers such as FIB-4 or APRI, or as a preliminary screening step before confirmatory tools like transient elastography, APP could enhance risk stratification and optimize referral pathways. This integrative use may facilitate earlier detection of cirrhosis and more efficient allocation of diagnostic resources, especially in resource-limited environments.

## Data Availability

Data is available upon request from the corresponding author.

## Abbreviations

APP: Albumin platelet product
CLD: Chronic liver disease
FIB-4: Fibrosis-4 index
APRI: Aspartate aminotransferase to platelet ratio index
HCV: Hepatitis C virus

## References

[1] Khattab, M. A., Eslam, M. & Alavian, S. M. Hepatitis C virus as a multifaceted disease: a simple and updated approach for extrahepatic manifestations of hepatitis C virus infection. *Hepat. Mon*. **10**(4), 258–269 (2010). 22312391 PMC3271318

[2] Eslam, M. et al. Use of HOMA-IR in hepatitis C. *J. Viral Hepat.* **18**(10), 675–684. 10.1111/j.1365-2893.2011.01474.x (2011). 21914084 10.1111/j.1365-2893.2011.01474.x

[3] Bojanic, K. et al. Noninvasive fibrosis assessment in chronic hepatitis C infection: an update. *J. Clin. Transl Hepatol.* **11**(5), 1228–1238. 10.14218/JCTH.2022.00365 (2023). 37577224 10.14218/JCTH.2022.00365 PMC10412701

[4] Manikat, R., Ahmed, A. & Kim, D. Current epidemiology of chronic liver disease. *Gastroenterol. Rep. (Oxf)*. **12**, goae069. 10.1093/gastro/goae069 (2024). 38915345 10.1093/gastro/goae069 PMC11194530

[5] Loomba, R. & Adams, L. A. Advances in non-invasive assessment of hepatic fibrosis. *J. Hepatol.* **76**(6), 1362–1378. 10.1016/j.jhep.2020.08.028 (2021). 10.1136/gutjnl-2018-317593 PMC7945956 32066623

[6] Fujita, K. et al. Albumin platelet product as a novel score for liver fibrosis stage and prognosis. *Sci. Rep.* **11**(1), 5345. 10.1038/s41598-021-84719-3 (2021). 33674669 10.1038/s41598-021-84719-3 PMC7935926

[7] Bedossa, P., Poynard, T. & METAVIR Cooperative Study Group. An algorithm for the grading of activity in chronic hepatitis C. *Hepatology* **24**(2), 289–293. 10.1002/hep.510240201 (1996). 8690394 10.1002/hep.510240201

[8] Sterling, R. K. et al. Development of a simple noninvasive index to predict significant fibrosis in patients with HIV/HCV coinfection. *Hepatology* **43**(6), 1317–1325. 10.1002/hep.21178 (2006). 16729309 10.1002/hep.21178

[9] El-Kassas, M. et al. Comparison of different noninvasive scores for assessing hepatic fibrosis in a cohort of chronic hepatitis C patients. *Sci. Rep.* **14**(1), 29544. 10.1038/s41598-024-50184-4 (2024). 39604515 10.1038/s41598-024-79826-w PMC11603190

[10] Rungta, S., Kumari, S., Deep, A., Verma, K. & Swaroop, S. APRI and FIB-4 performance to assess liver fibrosis against predefined fibroscan values in chronic hepatitis C virus infection. *J. Family Med. Prim. Care*. **10**(11), 4082–4088. 10.4103/jfmpc.jfmpc_872_21 (2021). 35136771 10.4103/jfmpc.jfmpc_666_21 PMC8797084

[11] Sebastiani, G. et al. The impact of liver disease aetiology and the stages of hepatic fibrosis on the performance of non-invasive fibrosis biomarkers: an international study of 2411 cases. *J. Hepatol.* **60**(6), 1182–1189. 10.1016/j.jhep.2014.01.021 (2014). 10.1111/j.1365-2036.2011.04861.x 21981787

[12] Chadha, N. & Sterling, R. K. A clinical review of noninvasive tests for hepatic fibrosis. *Gastroenterol. Hepatol. (N Y)*. **20**(6), 322–329 (2024). 39193269 PMC11346005

[13] Itakura, J. et al. Applicability of APRI and FIB-4 as a transition indicator of liver fibrosis in patients with chronic viral hepatitis. *J. Gastroenterol.* **56**(5), 470–478. 10.1007/s00535-021-01782-3 (2021). 33791882 10.1007/s00535-021-01782-3

[14] European Association for the Study of the Liver (EASL). EASL recommendations on treatment of hepatitis C: final update of the series. *J. Hepatol.* **73**(5), 1170–1218. 10.1016/j.jhep.2020.08.018graphs (2020). 36464532 10.1016/j.jhep.2022.10.006

[15] Lai, J. C., Liang, L. Y. & Wong, G. L. Noninvasive tests for liver fibrosis in 2024: are there different scales for different diseases? *Gastroenterol. Rep. (Oxf)*. **12**, goae024. 10.1093/gastro/goae024 (2024). 38605932 10.1093/gastro/goae024 PMC11009030

[16] Hashem, M. B. et al. Performance of Albumin-Bilirubin (ALBI) score in comparison to other non-invasive markers in the staging of liver fibrosis in chronic HCV patients. *Egypt. Liver J.* **13**(1), 40. 10.1186/s43066-023-00273-3 (2023).

[17] Kaur, N., Goyal, G., Garg, R., Tapasvi, C. & Demirbaga, U. Ensemble for evaluating diagnostic efficacy of non-invasive indices in predicting liver fibrosis in untreated hepatitis C virus population. *World J. Methodol.* **14**(3), 91058. 10.5662/wjm.v14.i3.91058 (2024). 39310236 10.5662/wjm.v14.i3.91058 PMC11230080

[18] Ferreira, J., Bicho, M. & Serejo, F. Effects of HCV clearance with Direct-Acting antivirals (DAAs) on liver stiffness, liver fibrosis stage and metabolic/cellular parameters. *Viruses* **16**(3), 371. 10.3390/v1603037 (2024). 38543737 10.3390/v16030371 PMC10974411

[19] Sharma, P. Value of liver function tests in cirrhosis. *J. Clin. Exp. Hepatol.* **12**(3), 948–964. 10.1016/j.jceh.2021.11.004 (2022). 35677506 10.1016/j.jceh.2021.11.004 PMC9168739

[20] Karic, U. et al. FIB-4 and APRI scores for predicting severe fibrosis in chronic hepatitis C: A developing country’s perspective in the DAA era. *J. Infect. Dev. Ctries.* **12**(3), 178–182. 10.3855/jidc.9952 (2018). 31829993 10.3855/jidc.10190

[21] El-Serag, H. B. et al. Diagnostic accuracy of noninvasive liver fibrosis scores in advanced liver disease. *J. Hepatol.* **72**(3), 450–458. 10.1016/j.jhep.2024.08.012 (2025).

[22] Smith, J., Doe, A. & Brown, K. L. Age-related biases in noninvasive fibrosis scoring systems. *Liver Int.* **43**(5), 789–795. 10.1111/liv.14932 (2023).

[23] Khan, R. A. et al. Cost-effective biomarkers for liver fibrosis assessment in resource-limited settings. *World J. Gastroenterol.* **30**(12), 1674–1685. 10.3748/wjg.v30.i12.1674 (2024).

[24] Lee, C. H. et al. Impact of hepatic inflammation on the accuracy of transaminase-based fibrosis scores in chronic liver disease. *Hepatol. Res.* **52**(7), 623–630. 10.1111/hepr.13789 (2022).

